# Evidence of homologous recombination as a driver of diversity in *Brachyspira pilosicoli*

**DOI:** 10.1099/mgen.0.000470

**Published:** 2020-11-11

**Authors:** Anish Pandey, Maria Victoria Humbert, Alexandra Jackson, Jade L. Passey, David J. Hampson, David W. Cleary, Roberto M. La Ragione, Myron Christodoulides

**Affiliations:** ^1^​ Molecular Microbiology, School of Clinical and Experimental Sciences, University of Southampton Faculty of Medicine, Southampton General Hospital, Southampton SO16 6YD, UK; ^2^​ Southampton NIHR Biomedical Research Centre, University Hospital Southampton NHS Trust, SO166YD, UK; ^3^​ Department of Pathology and Infectious Diseases, School of Veterinary Medicine, Faculty of Health and Medical Sciences, University of Surrey, Guildford, GU2 7AL, UK; ^4^​ School of Veterinary and Life Sciences, Murdoch University, Murdoch, Western Australia 6150, Australia

**Keywords:** *Brachyspira*genus, *Brachyspira pilosicoli*, pangenome, recombination, microbial evolution

## Abstract

The enteric, pathogenic spirochaete *Brachyspira pilosicoli* colonizes and infects a variety of birds and mammals, including humans. However, there is a paucity of genomic data available for this organism. This study introduces 12 newly sequenced draft genome assemblies, boosting the cohort of examined isolates by fourfold and cataloguing the intraspecific genomic diversity of the organism more comprehensively. We used several *in silico* techniques to define a core genome of 1751 genes and qualitatively and quantitatively examined the intraspecific species boundary using phylogenetic analysis and average nucleotide identity, before contextualizing this diversity against other members of the genus *Brachyspira *. Our study revealed that an additional isolate that was unable to be species typed against any other *Brachyspira* lacked putative virulence factors present in all other isolates. Finally, we quantified that homologous recombination has as great an effect on the evolution of the core genome of the *B. pilosicoli* as random mutation (*r*/*m*=1.02). Comparative genomics has informed *Brachyspira* diversity, population structure, host specificity and virulence. The data presented here can be used to contribute to developing advanced screening methods, diagnostic assays and prophylactic vaccines against this zoonotic pathogen.

## Data Summary

1. All samples sequenced and assembled during the course of this study have been deposited at the National Center for Biotechnology Information’s (NCBI’s) GenBank database under Bioproject PRJNA564276.
2. Metadata and GenBank accessions for the samples alongside those of other genomes used in analyses during this study are outlined in Table 1.

**Table 1.** Isolates used in this study and genome assembly metrics

* Brachyspira* species	Strain	Host	Location	Accession	GC (%)	Total length	# contigs	N50
* B. murdochii *	B11	Chicken	Australia	VYIL00000000*	27.70	3 156 632	26	528 032
* B. pilosicoli *	B04	Chicken	Australia	VYIM00000000*	27.92	2 607 200	11	1 319 479
* B. pilosicoli *	B06	Chicken	Australia	VYIN00000000*	27.81	2 789 651	80	165 358
* B. pilosicoli *	B12	Chicken	Australia	VYIO00000000*	27.91	2 593 600	9	2 586 710
* B. pilosicoli *	B14	Chicken	Australia	VYIP00000000*	27.91	2 588 857	47	181 709
* B. pilosicoli *	B31	Chicken	Australia	VYIQ00000000*	27.91	2 594 260	11	1 911 521
* B. pilosicoli *	B37	Chicken	Australia	VYIR00000000*	27.91	2 594 260	11	1 911 521
* B. pilosicoli *	SAP_774	Chicken	UK	VYIS00000000*	27.90	2 568 130	30	2 542 180
* B. pilosicoli *	SAP_822	Chicken	UK	VYIT00000000*	27.88	2 746 377	69	102 423
* B. pilosicoli *	SAP_859	Chicken	UK	VYIU00000000*	27.82	2 546 568	20	227 560
* B. pilosicoli *	SAP_865	Chicken	UK	VYIV00000000*	27.83	2 547 658	20	417 927
* B. pilosicoli *	SAP_894	Chicken	UK	VYIW00000000*	27.91	2 713 772	58	195 119
* B. pilosicoli *	SAP_898	Chicken	UK	VYIY00000000*	27.80	2 913 681	253	92 127
*B*. ?	SAP_772	Chicken	UK	VYIX00000000*	27.90	2 745 085	21	182 551
* B. aalborgi *	513	Human	Denmark	†	28.11	2 558 428	11	2 532 317
* B. hampsonii *	NSH-16	Pig	USA	CP019914	27.38	3 189 639	1	3 189 639
* B. hyodysenteriae *	ATCC27164	Pig	USA	NZ_CP015910	27.04	3 074 045	2	3 041 447
* B. innocens *	B256/ATCC29796	Pig	UK	ARQI01000129	27.73	3 281 611	130	52 799
* B. intermedia *	PWS/A	Pig	UK	CP002874	27.21	3 308 048	2	3 304 788
* B. murdochii *	DSM 12563	Pig	Canada	CP001959	27.75	3 241 804	1	3 241 804
* B. pilosicoli *	95/1000	Pig	Australia	CP002025	27.90	2 586 443	1	2 586 443
* B. pilosicoli *	B2904	Chicken	UK	CP003490	27.79	2 765 477	1	2 765 477
* B. pilosicoli *	P43/6/78	Pig	UK	CP002873	27.92	2 555 556	1	2 555 556
* B. pilosicoli *	WesB	Human	Australia	HE793032	27.73	2 889 522	1	28 899 522
* B. suanatina *	AN4859/03	Pig	Sweden	CVLB01000001	27.00	3 256 103	30	2 243 936

All *Brachyspira* genomes used during the course of this study are listed in this table. Isolates assembled during the course of this study are separated from assemblies sourced from public repositories by a dividing line and have the ‘*’ symbol following their accession number. The species of every assembly has been confirmed by Kraken and the accession numbers provided were obtained from NCBI GenBank. Genome statistics, including GC percentage, total length of the isolate genome, number of contigs constituting the assembly and the largest contig and the N50 value are given. N50 is a weighted median statistic that comments on the distribution of contig lengths and overall genome assembly quality. Fifty per cent of the entire assembly is contained in contigs equal to or larger than the N50 value. ‘B’ refers to the genus *Brachyspira*.

†The *B. aalborgi* strain was obtained from the Sanger METAHIT consortium (https://www.sanger.ac.uk/resources/downloads/bacteria/metahit/).

**Impact Statement**

The genus *Brachyspira* contains gut bacteria that can cause a disease called spirochaetosis in specific hosts. Among these organisms, *Brachyspira pilosicoli* is characterized by its unusually broad host range and its potential to cross from one species to another (zoonosis). However, there has been very little genomic data available for this organism. Our study is important for several reasons: (1) we introduced 12 newly sequenced draft genome assemblies to the literature, a 4-fold increase in the number of examined isolates; (2) we catalogued the intraspecific genomic diversity of the organism(s) comprehensively; (3) we found 1 isolate that was unable to be species typed against any other *Brachyspira *, and lacked putative virulence factors present in all other isolates, potentially suggesting a new species; (4) we quantified that homologous recombination has as great an effect on the evolution of the core genome of *B. pilosicoli* as random mutation. Overall, our in-depth characterization of the *Brachyspira* is important for understanding their genetic diversity, population structure, host specificity and virulence. All of these are important attributes that can contribute to efforts to develop new screening and diagnostic tools, and control measures such as vaccines.

## Introduction

*Brachyspira* (previously *Treponema *, *Serpula* and *Serpulina *) is the sole genus of the family *Brachyspiraceae* within the order Spirochaetales, phylum Spirochaetes [1]. The genus *Brachyspira* includes nine officially recognized species [2], several of which are pathogenic – primarily to pigs and poultry, although they can also infect other animals and humans. These species are flagellated, anaerobic, aero-tolerant Gram-negative spirochaetes that inhabit the large intestine, where they are intimately associated with the colonic or caecal mucosa. Infection with *Brachyspira hyodysenteriae* (classical agent), *Brachyspira hampsonii* or *Brachyspira suanatina* causes swine dysentery (SD), a severe colitis in pigs [3–5]. *Brachyspira murdochii* and *Brachyspira pilosicoli* also infect pigs, but cause milder colitis symptoms [6]. Infection of chickens with either *Brachyspira intermedia* or *Brachyspira alvinipulli* causes avian intestinal spirochaetosis (AIS) [7]. *Brachyspira innocens* is an enteric commensal of pigs, chickens and rats, and no particular disease has been associated with this species [8]. However, Burch *et al.* found that *B. innocens* infection was associated with poor performance in flocks and that this organism was significantly associated with below-target egg production in free-range flocks [9]. *Brachyspira aalborgi* was assumed to be the sole aetiological agent causing histologically identified intestinal spirochaetosis (IS) in humans (HIS) [10], but it was then reported that human gut colonization by *B. pilosicoli* is also common [11, 12].

*B. pilosicoli* is distinguished from the other *Brachyspira* species by its broad host range [2, 13] and zoonotic potential [13–16], and for being the sole causative agent of porcine intestinal spirochaetosis (PIS) [17]. *B. pilosicoli* can also infect chickens (causing AIS) [18, 19], wild ducks [20, 21], domesticated turkeys [22], pheasants [23, 24], rodents [25, 26], dogs [27–29] and horses [30], among several other animal species [2, 31]. Risk factors associated with human infection by *B. pilosicoli* include faecal–oral contamination and ingestion of water, living rurally and/or among animals, crowding, socioeconomic depression, travel to-and-from less economically developed countries and positive HIV status. A review collating global human clinical manifestations and prevalence of *B. pilosicoli* [2] cites that it is commonly found in faecal samples collected in the Middle East, Southeast Asia and rural Australia, but for example is less prevalent (~1.5 %) in urban parts of Australia [32], the UK and Belgium.

Comparative genomics provides an opportunity to investigate the diversity and interactions of pathogens circulating among domestic animal populations and humans. There is evidence of horizontal gene transfer taking place in the gut environment shaping both the phylum Spirochaetes and genus *Brachyspira *. Horizontal gene acquisition of auxiliary and secondary metabolism genes by phylum Spirochaetes from Gram-positive Firmicutes has been noted [33]. In addition, *B. hyodysenteriae* found in the porcine large intestine possess carbohydrate metabolism genes that are more similar to those of *Clostridia* spp. and *Escherichia coli* than to those of other spirochaetes [34]. Genomic characterization is important for understanding *Brachyspira* diversity, population structure, host specificity and virulence, and could contribute to developing advanced screening, diagnostic and control measures, including vaccines. The aim of our study is to contribute a better understanding of this potentially zoonotic pathogen by providing an in-depth genomic analysis of *B. pilosicoli* isolates.

## Methods

### Isolates, strains and samples

A total of 14 samples were isolated from chickens with AIS in Australia and the UK. *B. pilosicoli* isolates B04 and B06 were isolated in 1994, and B12 and B14, and B31 and B37 were all isolated in 1998: each pair was isolated from a different farm in South East Queensland, Australia. The farms were either broiler breeder farms or egg layers. The UK isolates SAP_774, SAP_822, SAP_859, SAP_865, SAP_894, SAP_898 and SAP_772 were collected from different farms in the UK between 2007–2011. All isolates were from different free range egg-layers. Of these, 13 were species designated by Kraken v0.10.5-beta [35] with the miniKraken database v20141208 to be *B. pilosicoli* and 1 – isolate B11 isolated in Australia – was a species designated as *B. murdochii *. These isolates were assembled as described below and are presented in Table 1.

Publicly available genus *Brachyspira* and *B. pilosicoli* reference strains were acquired from the Refseq and GenBank repositories using the ncbi_ftp_download script from bacs-genomics-scripts [36]. Confirmation of genus and species designation was done using Kraken. A total of 4 *B. pilosicoli* and 8 *Brachyspira* spp. genomes were sourced and are displayed in Table 1 alongside 40 publicly available *B. hyodysenteriae* genome assemblies in Data S1 (available in the online version of this article). The most complete draft genome (highest N50 value and lowest contig number) was selected for a given *Brachyspira* species where more than one was available.

### Microbiological identification prior to sequencing

#### α-glucosidase, β-glucosidase and α-galactosidase activity tests

Suspensions of *Brachyspira* in 0.1 M sterile phosphate-buffered saline (PBS) (≥McFarland 4.0) were prepared by transferring surface growth from FABA agar using a sterile swab. Aliquots of the suspensions were transferred into separate universal tubes and an α-glucosidase (p-nitrophenyl-α-d-glucopyranoside), β-glucosidase (p-nitrophenyl-β-d-glucopyranoside) or α- galactosidase (p-nitrophenyl-α-d-galactopyranoside) diatab (Rosco Diagnostics) was added to each aliquot. The suspensions were incubated anaerobically at 37 °C for 16 h. Results were recorded whereby a yellow colour change was regarded as positive and absence of colour change was regarded as negative for the respective enzyme activity.

#### Hippurate test

Suspensions of *Brachyspira* in 1 % (w/v) sodium hippurate solution (Sigma-Aldrich) (≥McFarland 2.0) were prepared by transferring surface growth from FABA agar using a sterile swab. The suspensions were incubated anaerobically at 37 °C for 24 h, after which 150 µl of API NIN (ninhydrin) reagent (BioMérieux) was added. Following 10 min incubation at room temperature, results were recorded whereby a blue-purple colour change was regarded as positive and a clear-orange colour change was regarded as negative for the ability to hydrolyse sodium hippurate to glycine and sodium benzoate.

#### Indole test

Suspensions of *Brachyspira* in brain heart infusion (BHI) medium supplemented with 10 % (v/v) foetal bovine serum (FBS) (≥McFarland 4.0) were prepared by transferring surface growth from FABA agar using a sterile swab. The inoculated BHI broth was incubated anaerobically at 37 °C for 24 h, after which 150 µl of API JAMES (Kovac’s) reagent (BioMérieux) was added. Following a 10 min incubation at room temperature, results were recorded whereby the formation of a pink-red pellicle was regarded as positive and a yellow pellicle was regarded as negative for the ability to cleave indole from tryptophan.

#### *Brachyspira* polymerase chain reaction (PCR)

Established genus *Brachyspira-* and species-specific PCRs were used for the initial identification of *Brachyspira* isolates. The genus-specific PCRs were based on the 16S rRNA gene and used to confirm that all the isolates were from the genus *Brachyspira* [37]. PCRs designed for the identification of *B. pilosicoli* were based on two well-conserved genes in the genus, the NADH oxidase (nox) and 16S rRNA genes [38] (Table 2). For PCR, a 20 µl reaction mixture was prepared consisting of GoTaq Master Mix (Promega), the forward and reverse primers (20 pmol µl^−1^) (Sigma-Aldrich), DNA template (20–50 ng µl^−1^) and DNAse-free water. PCR amplifications were performed using a Techne thermocycler as follows: 95 °C for 5 min to denature the DNA, followed by 30 cycles of denaturation at 95 °C for 60 s, annealing at 55 °C for 60 s and an extension at 72 °C for 60 s, followed by a final DNA extension at 72 °C for 7 min. Samples were then cooled to 4 °C.

**Table 2.** Primer sequences for genus *Brachyspira *- and species-specific PCR

Target species	Target gene	Primer name	Primer sequence (5′−3′)	Size (bp)	Reference
Genus *Brachyspira *	16S rRNA	Br16S-F	TGAGTAACACGTAGGTAATC	1309	[37]
		Br16S- R	GCTAACGACTTCAGGTAAAAC		
* Brachyspira * *pilosicoli*	16S rRNA	Acoli-F	AGAGGAAAGTTTTTTCGCTT	439	[38]
		Acoli-R	CCCCTACAATATCCAAGACT		

Genus *Brachyspira *-specific primers targeted the amplification of the 16S rRNA gene, which produced an amplicon of 1309 bp [37] (Table 2). *B. pilosicoli* species-specific primers targeting the 16S rRNA gene in *B. pilosicoli* produced an amplicon of 439 bp [38]. Gel electrophoresis of the PCR products was performed, and the species of each isolate was inferred using the amplicon sizes stated above and summarized in Table 2.

#### Growth, DNA extraction and sequencing

*B. pilosicoli* strains (and one accidental isolate of *B. murdochii *) were grown for 2 to 5 days on fastidious anaerobe agar (LAB090) supplemented with defibrinated sheep blood (5 % v/v) at 37 °C in an anaerobic gas jar with the Anaerogen gas-generating system (Oxoid, UK). Genomic DNA (gDNA) was extracted with the Wizard Genomic DNA Purification kit (Promega, USA) following the manufacturer’s protocol. Purified gDNA was quantified by absorbance with a NanoDrop UV-Vis Spectrophotometer 1000 (Thermo Fisher Scientific, USA) and with a Qubit 2.0 Fluorometer (Thermo Fisher Scientific, USA). The quality of all gDNA sample preparations was assessed by 0.7 % (w/v) agarose gel electrophoresis before sequencing. Isolates were sequenced at the Oxford Genomics Centre (https://www.well.ox.ac.uk/ogc/microbial-dna-sequencing/) on a MiSeq to generate 2×300 bp paired-end data.

#### Read quality control and genome assembly

Paired-end reads were assembled using the automated, A5-miseq pipeline v20160825 [39]. Briefly, adapter sequence removal was done using Trimmomatic v0.32 [40], followed by k-mer-based error correction using SGA v0.9.9 [41]. Contigs were assembled using the IDBA-UD algorithm v1.0.6 [42]. Misassemblies were detected and removed where read pairs were not mapping within an expected distance. A final, stringent round of scaffolding repaired broken contigs. Lastly, assembly metrics were determined using Quast v4.0 [43].

#### Multi-locus sequencing typing (MLST)

Designation of sequence types was done using Short-Read Sequence Typing 2 (SRST2) [44] and the *B. pilosicoli* MLST scheme [45].

#### Annotation, pangenome construction and phylogenetic inference

Genome assemblies were annotated with Prokka v1.12 [46], using the -use_genus flag and a list of proteins derived from three, previously annotated *B. pilosicoli* genomes with the -proteins flag. GFF annotations were used in conjunction with MAFFT v7.3.1 [47] as part of the Roary pipeline v3.8.0 [48] to generate the core genome alignment (gene presence based on blastp seq-ID: 0.95 and presence among 99 % strains) alongside the pangenome. The core alignment was used in conjunction with FastTree v2.1.11 [49], recompiled with -duse_double to resolve shorter branch lengths found between isolates with little variation. The generalized time reversible (-gtr) and nucleotide (-nt) model was used. Phylogenetic tree visualization and editing was done using Figtree v1.4.2 [50]. The pangenome was visualized alongside the phylogenetic tree using Phandango [51] (https://jameshadfield.github.io/phandango; date of accession: 19 July 2020).

#### Clusters of orthologous groups (COGs)

COGs were calculated by separating the core and accessory genome proteins and using the cdd2cog_script from bacs-genomics-scripts [36] to assign functional groups.

#### Recombination

*B. pilosicoli* assemblies were mapped to the closed genome of *B. pilosicoli* 95/1000 using Parsnp v1.2 [52] under default parameters. This generated a core genome alignment in XMFA format, which was converted to fasta format using the xmfa2fasta.pl script (https://github.com/kjolley/seq_scripts/blob/master/xmfa2fasta.pl; date of accession: 14 August 2020). The recombination analysis program ClonalFrameML (CFML) v1.11 requires both a fasta alignment and phylogenetic tree as inputs. A maximum-likelihood (ML) tree was created by converting the fasta alignment to phylip format using EMBOSS seqret v6.6 [53] and running PhyML v3.20180621 [54] on the phylip alignment using the HKY85 model. This process also calculated a transition/transversion ratio to be used as the -kappa input parameter in CFML. ML trees were converted to binary format using the R package ape v3.0 [55]. Bootstrapping (-emsim) was used until recombination parameter variance values were <10^−6^. Recombination was visualized on the core genome alignment using the ‘cfml_results.R’ script available in the ClonalFrameML github (https://github.com/xavierdidelot/ClonalFrameML; date of accession: 1 September 2020). Recombinant genes of interest to the study were found by extracting ‘importations’ detected by CFML and compared via blastx to the reference genome used to create the input core genome alignment. This identified genes that were functionally annotated by submitting the sequences to the Uniprot (discussed further below) and EggNOG V5.0 (http://eggnog5.embl.de/#/app/home) databases [56].

#### Average nucleotide identity (ANI)

ANI analyses were performed using FastANI [57], an orthology mapper utilized to calculate p-dist values (%), and the Python module PyANI [58] was used to infer and visualize ANI via multiple tests and heatmaps. These included (i) 1020 bp fragment blastn+ analysis, (ii) ANI-blast_all via legacy blastn on 1020 bp fragments (ANIb), (iii) MuMer alignment (ANIb/m) and (iv) tetranucleotide frequency analysis [59]. ANI was performed to distinguish isolate SAP_772 from the best representative genome assembly of the other eight *Brachyspira* spp. (*n*=8) as well as the newly assembled isolates of *B. pilosicoli* (*n*=12) and four reference genomes of *B. pilosicoli* (*n*=4).

#### Putative virulence factor dataset generation and clustering

A list of 231 putative virulence factors was created by combining and filtering replicate UniProt accession numbers from (i) a list of proteins with pathogenic and virulence potential [60] and (ii) a list of 26 mostly surface-exposed proteins identified as cross-reacting bands using immune pig sera [61]. The UniProt accessions were converted into coding sequence (CDS) nucleotide accessions from UniProt (https://www.uniprot.org/) and then downloaded using the batch entrez service (https://www.ncbi.nlm.nih.gov/sites/batchentrez). Both sets of identifiers are listed with full annotations and cluster identities in Data S2. The virulence factors in coding sequence format were converted into a format suitable for use with SRST2 by following the steps for generating a custom database (https://github.com/katholt/srst2#generating-srst2-compatible-clustered-database-from-raw-sequences). As part of the database clustering step, the database of 231 putative virulence factors was clustered into 207 putative virulence factors. This was due to sequences that shared a gene name and annotation despite having a sequence identity of less than 90 %. Gene names were edited manually with alphabetic characters in order to be recognized as alternate alleles by SRST2. For example, *arp_A, arp_B* were both annotated ‘*arp*’ but occupied separate sequence clusters). SRST2 v.1.8 was run with default parameters and used to map isolate read data (.fastq) against the virulence factor list to generate a list of (i) exact matches; (ii) >90 % coverage matches, where a minimum of 90 % isolate reads aligned to a virulence factor sequence, and the difference is given in SNPs and insertions/deletions (SRST2 interprets these matches as allelic variants of virulence factors); (iii) uncertain matches identified due to poor read depth, truncation or base ambiguity; and (iv) a lack of matches.

#### Statistics

General statistics (mean, standard deviation) were calculated using LibreOffice Calc v6.3.0 for Debian-based Linux systems. To determine which COG groups were over-represented in the core, accessory and pangenome, the composition of each COG category was examined in a 2×2 contingency table using the Chi-square test with twin-tailed *P*-value and Yate’s correction [62]. This was done using the GraphPad Prism calculator (https://www.graphpad.com/quickcalcs/contingency1.cfm, date of accession: 1 September 2020). A Bonferroni-adjusted *P*-value of 0.002 (0.05 *P*-value25/COG tests) was used as the significance threshold.

## Results

### Genome assembly

A total of 14 newly sequenced genomes that were all initially thought to be *B. pilosicoli* isolates were assembled. Subsequently, 12 of these were confirmed to be *B. pilosicoli,* 1 was identified as *B. murdochii* and 1 was an unknown *Brachyspira* species (SAP_772). The identity and source of the isolates, and the genome assembly quality control metrics such as GC content, length, contig number and N50 are shown in Table 1. Average *B. pilosicoli* genome length was 2 645 822 bp (+/−106 304) with a GC content of 27.87 % (+/−0.06). The 12 *B. pilosicoli* isolates were clustered into 8 sequence types (STs), based on a combination of 7 housekeeping loci (alleles of *adh*, *alp, est, gdh, glpK, pgm* and *thi* genes). We identified seven of these STs as novel (Table 3). This brings the total count of *B. pilosicoli* sequences type up to 94 STs.

**Table 3.** MLST allele data for 12 *B. pilosicoli* isolates

Isolate	*adh*	*alp*	*est*	*gdh*	*glp*	*pgm*	*thi*	ST
B04	5	**111**	**116**	12	**87**	**131**	**111**	**ST186**
B06	4	**107**	**112**	69	3	102	**103**	**ST187**
B12	5	37	105	27	18	49	35	ST134
B31	5	37	105	27	18	49	35	ST134
B37	5	37	105	27	18	49	35	ST134
B67	3	11	12	9	8	**132**	10	**ST182**
SAP_774	3	**108**	**113**	22	**88**	**133**	**112**	**ST183**
SAP_822	**55**	**109**	**114**	**68**	**89**	82	**113**	**ST184**
SAP_865	3	**110**	12	25	**90**	129	**114**	**ST188**
SAP_859	3	**110**	12	25	**90**	129	**114**	**ST188**
SAP_894	3	3	**115**	59	**91**	86	**115**	**ST185**
SAP_898	**55**	**109**	**114**	**68**	**89**	82	**113**	**ST184**

MLST allele data for the 12 *B. pilosicoli* isolates. Novel alleles and sequence types (STs) are indicated in bold. Allele abbreviations are as follows: *adh*, alcohol dehyrdogenase; *alp*, alkaline phosphatase; *est*, esterase; *gdh*, glutamate dehydrogenase; *glpK*, glucose kinase; *pgm*, phosphoglucomutase; and *thi*, acetyl-CoA acetyltransferase.

Isolate SAP_772 produced a good-quality draft assembly. The data from the quality control from the A5 pipeline revealed that an extremely high proportion (99.9 %) of reads passed the error correction and the contigs were kept to a low number compared to other *B. pilosicoli* assemblies. In addition to post-assembly read error correction, post-assembly quality control via Kraken revealed no unclassified contigs.

### *B. pilosicoli* pangenome analysis

A pangenome approach using the Roary pipeline provided a quantitative measure and insight into the shared genomic content of 12 study *B. pilosicoli* isolates and 4 reference *B. pilosicoli* isolates. The *B. pilosicoli* pangenome comprised 4590 genes. The core genome (present in 99–100 % of isolates tested) was 1751 genes. There were no genes present in the soft-core category (present in 95–99% isolates). The accessory genome (Data S3) consisted of the shell (1760 genes present in the genomes of 15–95 % of isolates tested) and the cloud (1079 genes present in the genomes of 1–15 % of isolates tested).

### COG analysis

**Fig. 1.**
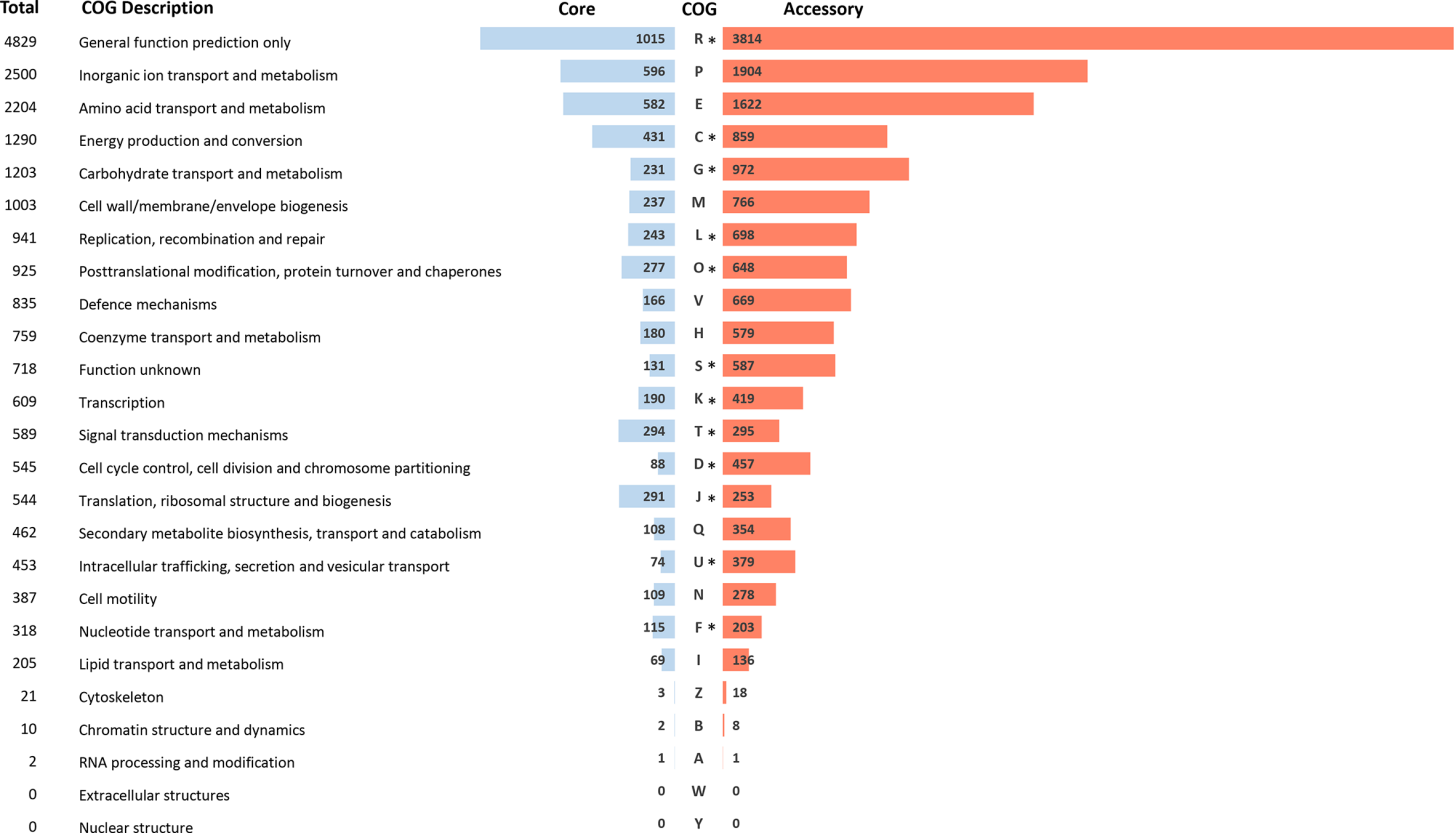
COG category metrics and proportional distribution in the pangenome of *B. pilosicoli.* The diagram shows the percentage proportion of COGs in the core (blue) vs accessory genomes (red). *, there is a significant difference in the distribution of the numbers of a category present in the core and accessory genome compared to COGs in the core/accessory not assigned to that given COG category. (Contingency table, χ^2^-corrected, 1 degree of freedom, twin-tailed, Bonferroni corrected *P* value<0.002.)

Distribution of COG categories was determined for the core and accessory genome (Fig. 1). The COG categories that were found most commonly among the *B. pilosicoli* isolates were inorganic ion transport and metabolism (COG P, *n*=2500), amino acid transport and metabolism (COG E, *n*=2204) and energy production and conversion (COG C, *n*=1290). Relative to the accessory genome, the *B. pilosicoli* core genome contained a higher proportion of genes involved with signal transduction and translation (COG T) and ribosomal structure and biogenesis (COG J). Of the 25 COG categories, 13 were present at significantly different distributions between the core and accessory genome compared to total COGs. These data are presented alongside statistics in Data S4.

### Phylogenetics

**Fig. 2.**
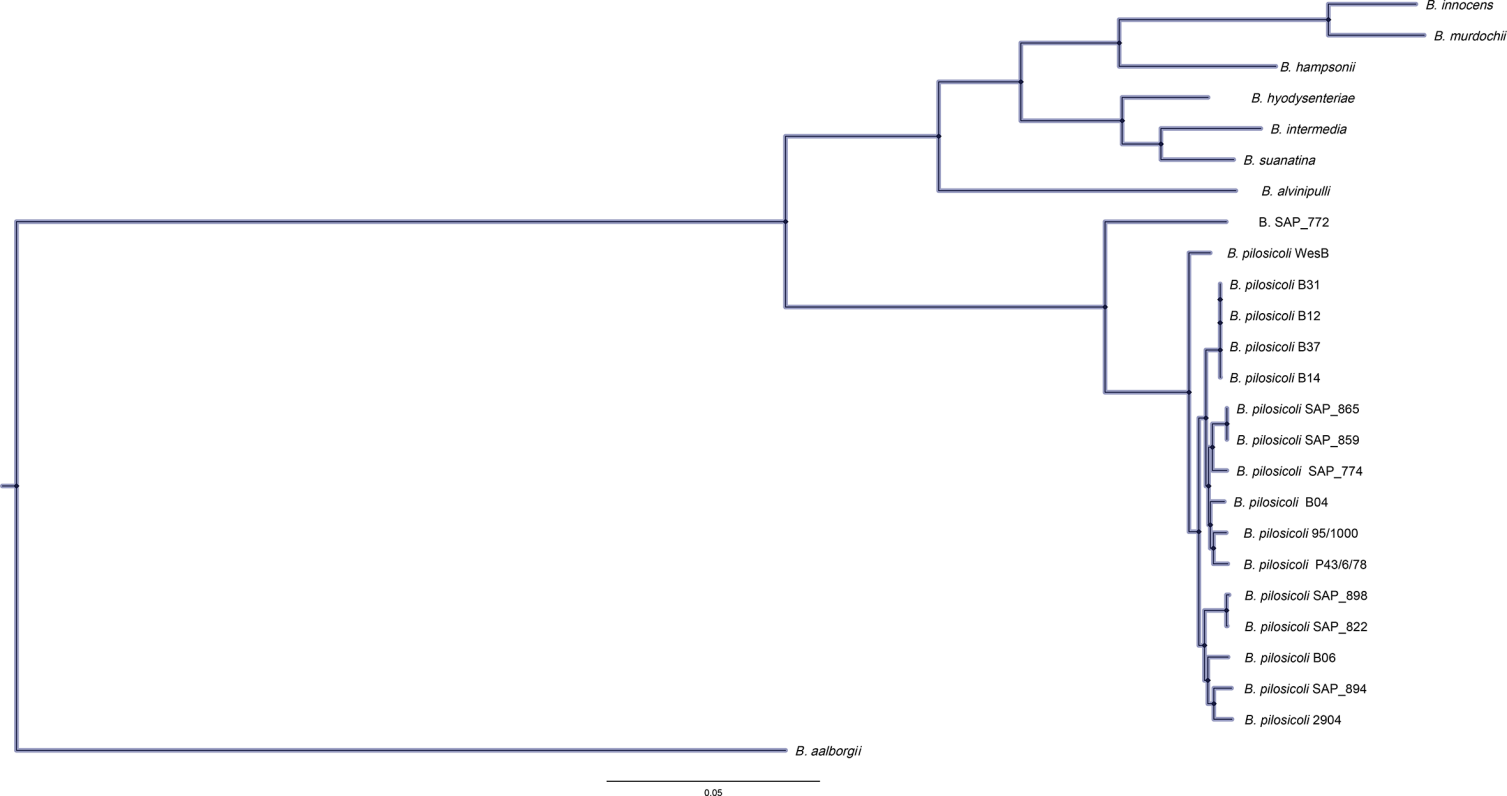
Phylogenetic tree. Phylogenetic inference reveals the extent of genomic diversity both within the genus *Brachyspira* and interspecifically among *B. pilosicoli* isolates. The tree was generated using RaxML version 8.2 and a GTR model. An alignment of 27 CDS was found to be core (95 % Seq-ID) to this dataset generated using the MAFFT aligner, rooted with *B. aalborgi* genome, which was found to be the most distant.

A core genome alignment (99 % gene coverage among strains defined as core) was generated via Roary for the genus *Brachyspira *. Based on a core alignment of 9393 sites (27/21395 total genes), *Brachyspira* isolate SAP_772 occupied one branch (red), but still clustered closer to *B. pilosicoli* than any other species (Fig. 2). Among the genus *Brachyspira *, *B. innocens* and *B. murdochii* were found to group together, as did *B. intermedia* and *B. suanatina* (blue). Rooting by the lowest common ancestor (Spirochaete: *Brevinema andersonii *, based on 16S rRNA phylogeny of bacteria and archaea strains) was not possible owing to the loss of tree resolution following its inclusion.

### Recombination

Recombination analysis was performed on the *B. pilosicoli* core genome (*n*=16 isolates, *n=*1751 genes). These metrics included the relative rate of recombination to mutation: R/theta=0.38+/−5.9E^−6^, the mean length of detected recombinant regions: 1/delta=1.9E^−2^+/−1.5E^−8^ and mean divergence level between recipient and donor: Nu=5.1E^−2^+/−4.0E^−8^. The parameters were multiplied together to calculate the relative effect of recombination compared to the relative effect of mutation (*r*/*m*=1.02). The metrics revealed that while homologous recombination occurred approximately one-third (0.38: R/theta) as often as mutation, the overall effect of recombination (in introducing diversity) on the core genome of *B. pilosicoli* was approximately the same as the effect of mutation (*r*/*m*=1.02). To make a broad comparison, 40 public genome assemblies of *B. hyodysenteriae* were analysed similarly. Here, the R/theta was 0.81 and the resultant *r*/*m* value was 3.99. The visualization showed that recombination events were found scattered throughout the phylogeny of *B. hyodysenteriae* (Data S5).

**Fig. 3.**
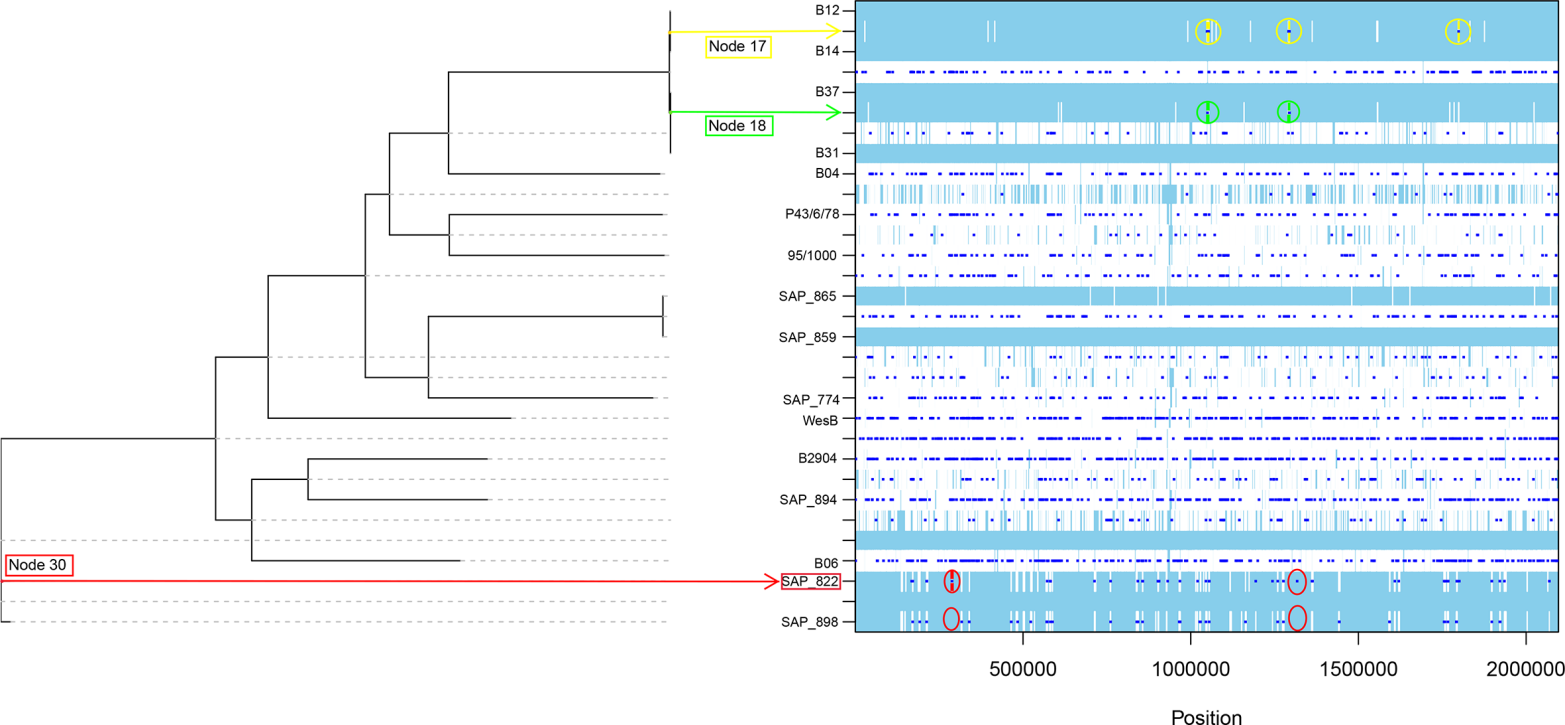
Recombination detection on the core genome of *B. pilosicoli.* Recombination events and sites are indicated along the branches of the phylogenetic tree. A given branch may display results for a specific isolate (see isolate label) or for an ancestral phylogenetic node (no isolate label). Recombination events are indicated by dark blue bars, while light blue sites are used to indicate no substitution and white sites indicate that a convergent mutation has occurred (one base or more) at that point in the phylogeny. When multiple convergent events occur within short nucleotide distance of each other a recombination event or ‘importation’ is identified by the software at a specific position in the core alignment affecting *x* number of isolates or *x* number of ancestral phylogenetic nodes which affect multiple isolates. Recombination events discussed in the Results section are indicated with yellow, green and red circles. Their corresponding genes are identified with full annotation data in Data S6.

Recombination events were detected frequently throughout the core genome phylogeny of *B. pilosicoli* but were not universally distributed among every isolate examined (Fig. 3). Among closely related isolates, recombination analysis can identify recombination events (specifically, ‘importations’ of DNA bases that do not match the clonal genealogy). These data can be used to identify the genes affected by this allelic variation or novel gene gain that separates one isolate from another isolate(s) or group of isolates on a node from another.

Data S6 shows that nine putative recombination events were detected in five genes, i.e. amidotransferase; *gatA* (alanine cation symporter); *alsT* (amino acid carrier); *cpdB* (nucleotidase); hypothetical proteins hp1 (putative growth rate regulator) and hp2 (putative cell division activator). These events were detected in the ancestral node separating ST 134 isolates B12 and B14 from B37. Furthermore, two importations were detected among another *alsT* gene and a hypothetical protein (no obvious function) in the node separating B37 from B31. In addition, recombination events were found in ST 184 isolates indicating gene gain in isolate SAP_822. These importations of DNA were (i) absent in recombination events and (ii) absent of convergent mutation in SAP_898 at the same or similar position in the core genome alignment. While both genes gained were hypothetical with unknown function in the COG database, Hp 4 contained DUF3298 domains that are also found in peptidoglycan deacetylase proteins such as PdaC of *Bacillus subtilis *. Interestingly, of all the genes, Hp 5 was found to be a spirochete-specific oxidoreductase containing VMA domain (a potential ‘intein’ or self-splicing, parasitic, mobile domain). Homologues of this protein are found in a select group of enteric pathogens that includes *B. intermedia *, *B. hampsonii* and *Treponema* spp*.*

Comparing the *B. pilosicoli* phylogenetic tree, before and after recombination correction, revealed an alternated phylogeny not just with regards to the branch lengths but also to the topology of the tree (Data S7). Accounting for recombination resulted in the generation of a new ancestral node in the phylogenetic tree, revealing that isolates SAP_822 and SAP_898 did not share the same branch as indicated previously.

### Discovery of a potential new species similar to *B. pilosicoli*

**Fig. 4.**
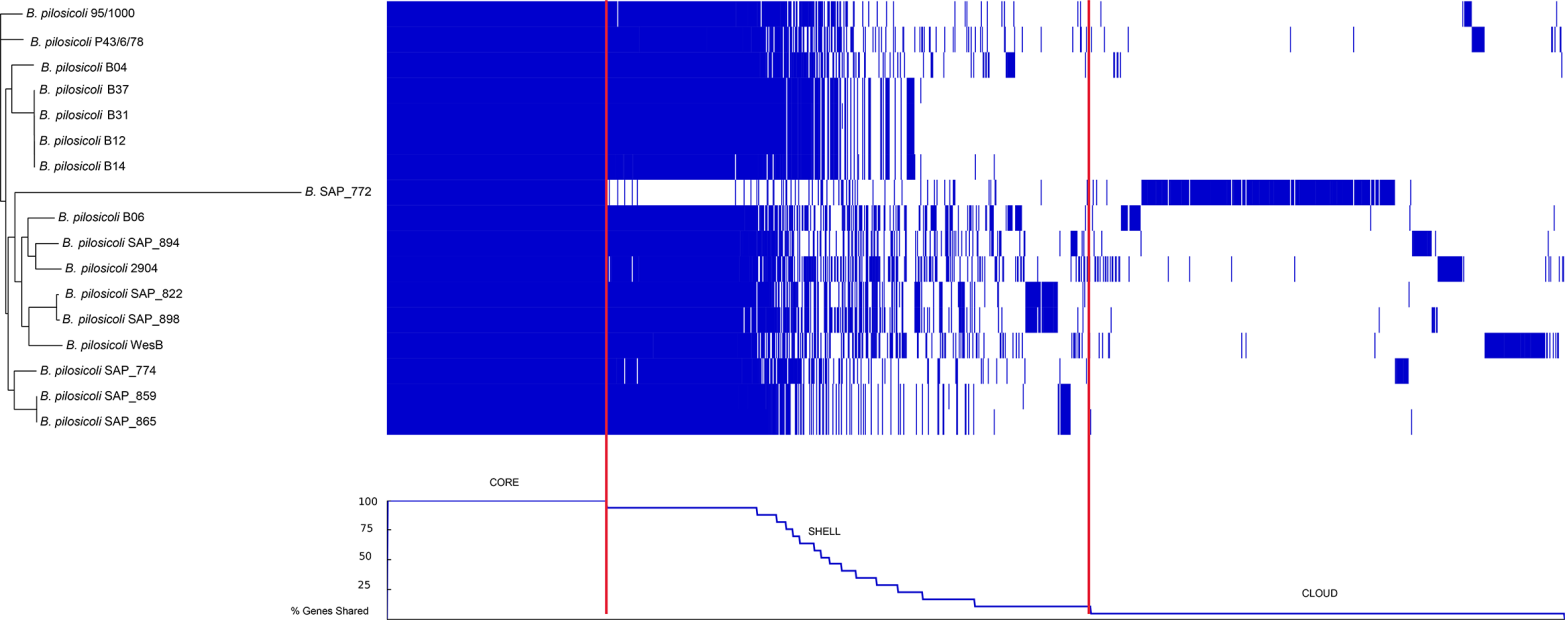
Gene distribution map of the *B. pilosicoli* pangenome plus *Brachyspira* SAP_772. On the left of the figure is an unrooted, ML phylogeny based on an accessory genome alignment (for more accurate inference of phylogenetic relationships see Fig. 2). In the centre, blue segments represent gene presence and white segments represent gene absence. The pangenome is displayed, starting from the core genome on the left and transitioning into the accessory genome (shell and cloud genomes) with increasing gene sequence disparity. The bottom graph displays a trace showing the percentage of isolates containing blue segments of gene presence. Similar to the centre, the graph starts with the core genome (*n*=17 genomes) and falls steadily as it transitions into the accessory [shell: *n*=2–16 isolates (approximately 95 % - >1 % of the *B. pilosicoli* plus B. SAP_772 cohort) and cloud: *n*=1 isolate, approx. <1 % of the cohort) genome displaying blocks of genes shared by fewer and fewer genomes. Red lines are used to mark the transitions from the core genome to the shell and cloud genomes.

In the primary analysis of the pangenome data, an anomalous result was seen by the inclusion of isolate SAP_772, which was originally thought to be a *B. pilosicoli* based on Kraken species designation. Thus, SAP_772 was excluded from any analysis related to the *B. pilosicoli* core genome. In comparison to the data mentioned above, the *B. pilosicoli* pangenome including SAP_772 expanded the total gene count from 4590 genes to 5772 genes spread across the now 17 isolates tested. Core genome size decreased from 1751 to 983 genes and the accessory genome (shell+cloud) increased in size. Again, no genes were identified as belonging to the soft-core genome category (the genes present in 95–99 % of isolates tested). The pangenome is displayed qualitatively in Fig. 4 and confirms that gene content distinguishes *B. pilosicoli* SAP_772 from the rest of the isolates tested, with large portions of the genome that would otherwise be core, missing from the isolate. The accessory genome was quantified (Data S3), and this revealed that *B. pilosicoli* SAP_772 possessed the greatest allelic and genomic diversity, with 1197 novel genes and/or gene variants. This diversity was ~3.5-fold greater than the next most diverse isolate (reference strain *B. pilosicoli* WesB, with 343 novel gene and/or allelic variants) and 5-fold greater than the third most diverse isolate (reference strain *B. pilosicoli* 2904, with 220 novel gene and/or allelic variants).

**Fig. 5.**
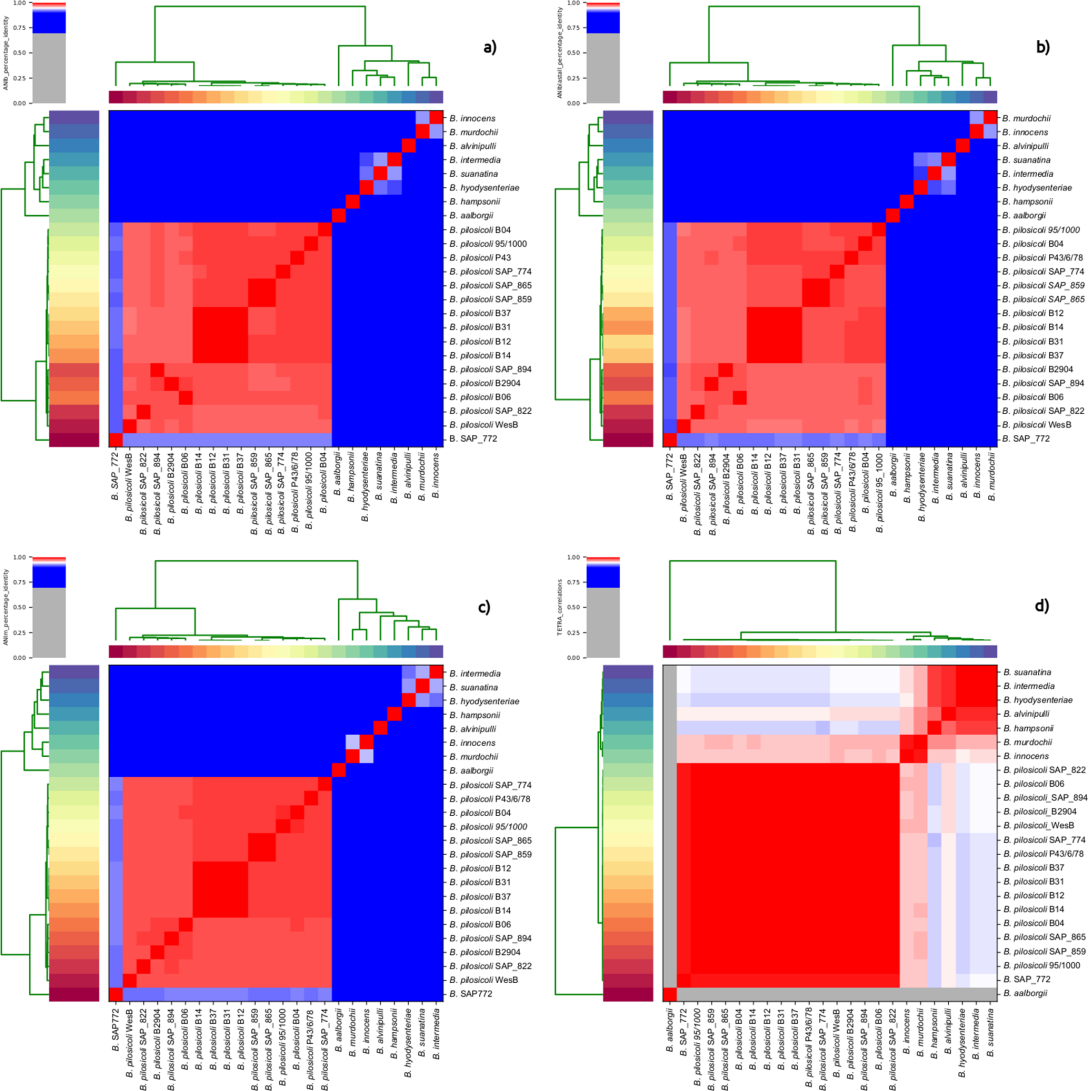
Average nucleotide identity (ANI). These four heatmaps visualize four tests performed by PyANI. These are (a) 1020 bp fragment blastn+ analysis, ANIb (b) ANI-blast_all via legacy blastn on 1020 bp fragments, ANIb-allvall, (c) muscle alignment ANI-M and, lastly, (d) tetranucleotide frequency analysis ANI-TETRA. As shown by the scale, red indicates increasing homology, while blue denotes decreases in homology between isolates examined in the heatmaps.

To discriminate the intraspecific genetic diversity between isolate SAP_772 and other *Brachyspira* spp. isolates, a cohort was created where the genomes of confirmed *B. pilosicoli* isolates (*n*=16; 12 newly assembled and 4 publicly sourced) was combined with 8 representative genomes of the other *Brachyspira* species. This cohort was subjected to species delineation using whole-genome average nucleotide identity (ANI). This metric demonstrates a percentage of the average nucleotide matches among all orthologous genes between two or more comparator genomes. An ANI of >95 % is indicative of the interspecific border. FastANI analysis (Data S8) showed that isolate SAP_772 had an average ANI of 80.31 %+/−1.43 when compared to the genus *Brachyspira *, and an average ANI of 91.63 %+/−0.09 when compared to *B. pilosicoli.* PyANI was used to calculate ANI and visualize the data via heatmaps using four experimental methods (Fig. 5). In three out of four analyses (ANI-muscle, ANI-blast and ANI-blast_all vs all, Fig. 5a–c) PyANI confirmed the FastANI results, i.e. that SAP_772 was divergent from the rest of the *Brachyspira* spp., and although more related to *B. pilosicoli *, was beyond the boundary for inclusion in this species. In the final method, tetranucleotide analysis (ANI-Tetra, Fig. 5d), SAP_772 nested amongst the *B. pilosicoli* isolates.

### Detection of genes encoding putative virulence factors

All study isolates (*n*=12 *B. pilosicoli* isolates plus *B.* SAP_772) were screened for the presence of putative virulence factors using SRST2 and a curated list of 207 previously identified genes (Data S2). The raw data are presented in Data S9 and are quantified in Table 4, which shows the number of matches (exact matches plus matches within 90 % sequence identity) alongside an absence of matches. In this screening, 11 out of 12 of the *B. pilosicoli* isolates displayed exact matches or putative allele assignment of 196.6+/−3.9 out of a total of 207 virulence factors. One strain of *B. pilosicoli* (B14) had fewer matches than the other *B. pilosicoli* isolates, but still had more than isolate SAP_772. Isolate SAP_772 both (i) showed matches between approximately 160 virulence factors and (ii) had the greatest paucity of matches (*n*=16 virulence factors). Undetected matches indicate that <90 % of the gene in question was covered by the sequence reads, exceeding the threshold for either an exact match or putative allele sharing >90 % sequence identity. Instances where an undetected match was unique to a given isolate are detailed in Table 5. This indicates that isolate SAP_772 has 10 undetected putative virulence factors that are otherwise represented among the rest of the *B. pilosicoli* isolates. Lastly, there were nine putative virulence factors that were undetected in multiple isolates, which are displayed in Data S10. These include CDS B204_orf2390 (basic membrane lipoprotein); BP951000_1477 (phage capsid protein); BP951000_2112 (peptidase); *bspA* (membrane surface antigen); *gmhB* (lipooligosaccharide (LOS) biosynthesis phosphatase); *lepB* (signal peptidase I); *rfbF* (LOS biosynthesis cytidyltransferase); *vsh* (phage capsid protein); and *vspD* (variable surface protein).

**Table 4.** Matches and no matches to *B. pilosicoli* and *B.* SAP_772 isolates subject to virulence factor analysis

Sample	Matches/ putative alleles	No match
* B. pilosicoli* B12	200	3
* B. pilosicoli* B31	200	4
* B. pilosicoli* B37	199	4
* B. pilosicoli* SAP_774	199	4
* B. pilosicoli* B04	198	6
* B. pilosicoli* SAP_859	198	4
* B. pilosicoli* SAP_865	197	4
* B. pilosicoli* B06	196	7
* B. pilosicoli* SAP_898	194	3
* B. pilosicoli* SAP_894	190	9
* B. pilosicoli* SAP_822	188	10
* B. pilosicoli* B14	171	6
*B.* SAP_772	159	16

This table lists the lack of matches versus exact matches and instances of putative allele assignment using SRST2 default parameters. Exact matches are instances where sequencing reads have mapped in perfect alignment with a virulence factor CDS. SRST2 assigns putative allele status to any sequence sharing a minimum of 90 % coverage with reads mapping to it. Uncertain results flagged by the pipeline were inspected manually and removed from counts.

**Table 5.** List of putative virulence factors undetected among one sample of isolate reads from *B. pilosicoli* or *B.* SAP_772

Isolate	No.	Undetected putative virulence factors
SAP_772	10	B2904_orf1521, iron/sulphur flavoprotein
		BP951000_1807, membrane lipoprotein
		*murD*, member of the *mur* operon of lipooilgosaccharide (LOS) biosynthetic modification ligases and synthases
		*pldB,* phospholipase
		*rfaD,* LOS biosynthetic epimerase
		*tolR,* outer-membrane biopolymer transport protein
		arp_G*, ankyrin repeat-containing protein
		batA_B*, aerotolerance-related membrane protein
		mcpB_H*, methyl-accepting chemotaxis protein B
		mcpB_K*, methyl-accepting chemotaxis protein B
SAP_894	3	BP951000_0437, peptidase C14, caspase catalytic subunit p20
		BP951000_1159/*pfpI*, family intracellular peptidase
		*ftsH,* ATP-dependant zinc metalloprotease
SAP_822	3	ADK31727/*pep*, peptidase
		BP951000_0437, peptidase C14, caspase catalytic subunit p20
		BP951000_1779, probable metal-dependent glycoprotease
B06	2	B2904_orf2005, lipoprotein
		B2904_orf651, lipoprotein
B14	1	pep_A*, peptidase
SAP_774	1	BP951000_2039, putative periplasmic binding protein
B04	0	
B12	0	
SAP_859	0	
SAP_865	0	
B31	0	
SAP_898	0	
B37	0	

Accession numbers, cluster identities and functional annotation available for putative virulence factors are detailed in Data S1. *These proteins had the same functional annotation as another protein named the same but possessed <90 % sequence identity. They are labelled with an underscore and capital letter to distinguish them for virulence factor screening.

## Discussion

To our knowledge, our study is the most comprehensive genomic analysis of *B. pilosicoli* to date and gives insight into the intraspecific genomic diversity of this organism. *Brachyspira* species have a single circular chromosome varying in size from ~2.5 to 3.3 Mb, with a similar G+C content of ~27 %, each encoding for >2300 proteins [4, 5, 34, 60, 63–69]. Close similarities were observed in the 16S rRNA gene sequences of most species, which implies relatively recent speciation events [70]. *B. pilosicoli* has the second smallest genome (~2.6 Mb) of all *Brachyspira* species reported and has no described extra-chromosomal elements, with only *B. aalborgi* being marginally smaller. Nevertheless, *B. pilosicoli* strains, taken together, encode a greater diversity of proteins than other *Brachyspira* species, which is possibly attributable to a greater number of gene duplications than other species of the genus [2, 60, 66, 70].

A previous study [60] compared *B. pilosicoli* strain 95/1000 with *B. hyodysenteriae* strain WA1 and *B. murdochii* strain 56–150 and found that the smaller genome of *B. pilosicoli* 95/1000 contained more genes than the other species in the COGs for cell motility (COG-N), intracellular trafficking, secretion and vesicular transport (COG-U), energy production and conversion (COG-C), co-enzyme transport and metabolism (COG-H) and lipid transport and metabolism (COG-I). From our study, the greater diversity of genes involved particularly in energy production and conversion (COG C) in the *B. pilosicoli* isolates agrees with this prior analysis.

Phylogenetic inference coupled with ANI suggests a close relatedness between *B. murdochii *, *B. innocens* and *B. suanatina,* as well as between *B. intermedia* and *B. hyodysenteriae. B. pilosicoli* and *B. aalborgi* stand apart, with the latter remaining the most phylogenetically distant from all other *Brachyspira* species. This observation is supported by previous studies using DNA–DNA hybridization or whole-genome comparison, which also found a high level of homology between *B. murdochii *, *B. intermedia *, *B. innocens* and *B. hyodysenteriae* [60, 71, 72].

In contrast to the clonal population structure of other *Brachyspira* species (the sole exception so far being *B. aalborgi* [66]), the population structure of *B. pilosicoli* is thought to be driven by high levels of recombination [16, 66, 70]. This observation has been based on multilocus enzyme electrophoresis [73], pulsed-field gel electrophoresis [74], MLST [16] and variable-number tandem repeat analysis [75]. Through the higher resolution whole-genome analysis, we detected a plethora of recombination events spread throughout the core genome of *B. pilosicoli.* Principally, we found that isolates phylogenetically clustered according to location data: an exception to this was *B. pilosicoli* B06, which was found to be genetically more similar to isolates sourced from the UK poultry farms, rather than from the Australian farms. This was confirmed in both genus-specific and species-specific phylogenetic comparisons. This may be either an example of a common strain-specific evolution in an avian enteric pathogen, or maybe an adaptation of isolates found in egg-laying chickens. The UK chickens in this study only included egg-laying birds and not broiler birds. This makes sense from an evolutionary point of view, as the species has the potential to inhabit and infect multiple hosts and may therefore import a variety of exogenous DNA from other species found within these varied environments. While recombination and substitution events were detected in most isolates examined and in every ancestral node of the phylogenetic tree, a paucity of these events was seen when comparing isolates SAP_859, SAP_865, B37, B31, B14 and B12. These data, combined with low accessory genome size and extremely high ANI (when comparing the isolates within both groups), suggested evidence of gene loss and gain events and/or allelic variation specific to isolates all within the same sequence type. A recent study acknowledged that besides introducing diversity, homologous recombination acted as a cohesive force on population structure among prokaryotic species with >95 % ANI and a minimum *r*/*m* value of 0.25 [76].

Correcting for recombination in the phylogenetic inference of *B. pilosicoli* revealed not only corrections to the branch lengths, but also to the topology of the tree, as two isolates (SAP_898 and SAP_822) were seen to diverge from a common ancestor, rather than occupying the same branch. Our study inferred an *r*/*m* value of 1.02, based upon a core genome alignment of >2 000 000 sites, which was based upon mapping 15 isolates to the *B. pilosicoli* 95/1000 reference genome. The *r*/*m* metric infers that recombination is just as likely as mutation to have an impact upon the evolution of the core genome of this organism. This value is 5 times greater than that previously calculated for the genus *Brachyspira* [77], although this estimate was based on 7 housekeeping loci across 36 *Brachyspira* spp. strains. A comparison of *r*/*m* values of multiple bacterial genera found *Brachyspira* to generate one of the lowest signals [78]. To our knowledge, no other study has studied genome-wide recombination for this species. *B. pilosicoli* does not have the highly recombinogenic activity of organisms such as *Neisseria* spp*.* [78] and it is not as clonally structured as *B. hyodysenteriae* and *B. intermedia* [16].

Through the introduction of novel and varied fragments, homologous recombination was thought to be responsible for shaping the evolution of the zoonotic pathogen *B. pilosicoli *, in contrast to the more clonal population structure of other *Brachyspira* spp. such as *B. hyodysenteriae* and *B. intermedia* [16, 72]. Our study also revealed a dynamic impact on horizontal gene transfer events, affecting the core genome of *B. hyodysenteriae* to a far greater degree than *B. pilosicoli.* This is likely because *r*/*m* is always calculated relative to a sample. A *B. hyodysenteriae* cohort with double the isolates and greater inter-sample phylogenetic diversity gave the potential of many more sites in the core alignment analysed. This likely resulted in the greater detection of importations of novel DNA relative to *B. pilosicoli. B. hyodysenteriae* has a history of horizontal gene transfer events with (a) phage-like virulence plasmids and (b) bacteriophage between *B. hyodysenteriae* and phylogenetically related members of the *Brachyspira* such as *B. intermedia* and *B. murdochii* [63].

Our study revealed an additional isolate that was unable to be species typed against any other *Brachyspira* and suggests a possible new species that is related to, yet distinguishable from, *B. pilosicoli *. This was indicated by the high accessory genome content of isolate SAP_772 compared to *B. pilosicoli* isolates and by genus-wide phylogenetic inference. SAP_772 was further examined using ANI, which is a quantitative way of defining the species boundary and a well-characterized *in silico* substitute for DNA–DNA hybridization [59]. ANI was used to quantify the distance within the genus *Brachyspira* and between/within *B. pilosicoli.* Organisms that belong to the same species have an ANI value of >95 % and this was estimated using multiple methods and two pipelines. *Brachyspira* isolate SAP_772 was found to be similar to *B. pilosicoli,* compared to the rest of the *Brachyspira* spp., by FastANI, phylogenetic inference and three of the four PyANI methods (ANI-muscle, ANI-blast and ANI-blast_all). However, despite this similarity, the ANI scores and visualizations suggest SAP_772 falls outside the ANI boundary for a species (ANI ≤95 %), whereas all other *B. pilosicoli* isolates were within this boundary in every ANI testing method used. One PyANI method, tetranucleotide correlation, showed isolate SAP_772 nesting within *B. pilosicoli *. However, this analysis also resulted in *B. suanatina *, *B. intermedia* and *B. hyodysenteriae *, and *B. alvinipulli* and *B. hampsonii* clustering together. This may indicate a paucity of resolution when using the tetranucleotide correlation with these five *Brachyspira* species.

Furthermore, our study generated an indexed, clustered database of 207 putative virulence factors based on proteins identified in previous studies [60, 61]. On average, the *B. pilosicoli* isolates harboured 196 matches or putative allele assignments. This indicated that the dataset was relatively representative of *B. pilosicoli* as a species and warranted investigation into putative virulence factors that were undetected. Isolate SAP_772 displayed both a reduced virulence factor match score and an increased number of no matches compared to the confirmed *B. pilosicoli* isolates. In particular, our data indicate that SAP_772 lacks a large number of outer-envelope, LOS biosynthesis genes, the presence of which has displayed serological heterogeneity among *B. pilosicoli* strains [79]. The reduced virulence factor score is most likely due to the presence of allelic variants of the ‘absent’ *B. pilosicoli-*specific VF panel. Taken together, this evidence suggests isolate SAP_772 as a candidate for full phenotypic characterization to support a potentially novel species designation.

In summary, the contribution of new *B. pilosicoli* isolate genomes generated during the current study has allowed us to quantify and visualize the impact of homologous recombination as a force importing DNA on the core genome of *B. pilosicoli* for the first time. The *in silico* discovery of a potentially novel species represented by an isolate similar to *B. pilosicoli *, but lacking putative virulence factors, suggests potentially greater diversity among the genus *Brachyspira* than previously reported. The identification of a more robust core genome, alongside screening for common putative virulence factors, will aid researchers in developing a vaccine against an organism that is responsible for morbidity among livestock and has the potential for zoonotic transmission amongst a variety of species including pigs, poultry, wildlife species, dogs and humans [66].

## Supplementary Data

Supplementary material 1
